# Humic-like Substances Extracted from Hydrochar as an Additive for Foliar Fertilization in Tomato Plants

**DOI:** 10.1021/acsomega.5c05053

**Published:** 2025-08-26

**Authors:** Suelen A. Mondek, Isabela C. Constantino, Odair P. Ferreira, Márcia C. Bisinoti, Emilio C. Miguel, Thaiz B. A. R. Miguel, Gustavo Metzker, Mauricio Boscolo, Altair B. Moreira

**Affiliations:** † Institute of Biosciences, Humanities and Exact Sciences, Department of Chemistry and Environmental Sciences, São Paulo State University (UNESP), 15054-000 São José do Rio Preto, São Paulo, Brazil; ‡ Department of Chemistry, Laboratory of Advanced Functional Materials (LaMFA), State University of Londrina (UEL), 86055-900 Londrina, Paraná, Brazil; § Department of Metallurgical Engineering and Materials (DEMM) and Analytical Center, Biomaterials Laboratory (BIOMAT), Federal University of Ceará (UFC), 60440-900 Fortaleza, Ceará, Brazil; ∥ Food Engineering Department, Biotechnology Laboratory, Federal University of Ceará (UFC), 60440-900 Fortaleza, Ceará, Brazil

## Abstract

Biomass reuse and the development of fertilization strategies are approaches for enhancing agricultural productivity. Humic-like substances (HLS), extracted from hydrochars of various biomasses, exhibit valuable characteristics such as diverse organic composition and nutrient complexation, which can stimulate plant growth. However, few studies have investigated HLS as a foliar fertilization application. In this work, HLSs were extracted from two hydrochars derived from sugar cane bagasse and vinasse: HLS_A (additive H_3_PO_4_) and HLS_B (additive KOH), which were then characterized. HLSs were applied by foliar spraying at 50, 100, and 300 mg of TOC L^–1^, with and without fertilizer in tomato plants (*Solanum lycopersicum* L.). Plant development was monitored for 35 days, and growth, chlorophyll (ICF), and micromorphological evaluation (SEM) measurements were recorded. Both HLSs influenced plant growth. HLS_A at 100 mg of TOC L^–1^ with fertilizer increased shoot length, dry biomass, and chlorophyll content by 18, 20, and 13.9%, respectively, compared to the control. Similarly, HLS_B at 300 mg of TOC L^–1^ showed comparable increases in shoot length (17.4%) and dry biomass (15%) but was more effective in enhancing chlorophyll index (28.2%). Plants treated with HLS_B also showed an increase in root length and root dry weight by 25%, respectively. Treatments using only HLS also produced positive results, suggesting HLS's intrinsic stimulating effects and without signs of anatomical ruptures in the cellular structure. The findings indicate that both HLS are promising for use in sustainable agriculture, endorsing the reuse of sugar-energy industry residues.

## Graphical abstract

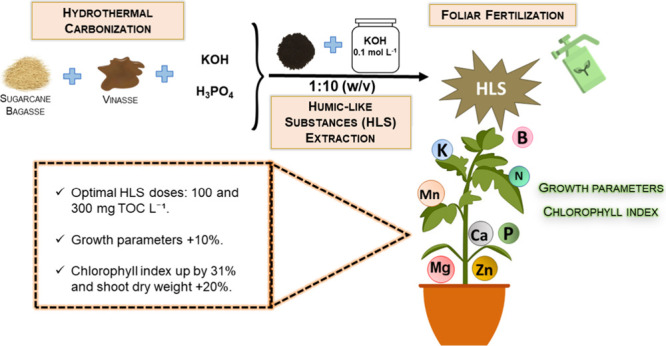

## 1 Introduction

The need to increase food production in a sustainable manner has become a central concern in global agricultural policy, particularly within the framework of the Food and Agriculture Organization (FAO)^1^ and United Nations Sustainable Development Goals (SDGs). These goals promote global strategies to combat hunger through sustainable agriculture (SDG 2) and waste reuse (SDG 12).^2^ To achieve these goals, more efficient technologies, such as controlled-release fertilizers,^3^ biostimulants,^4^ and the utilization of waste from different agricultural production chains, are becoming increasingly essential to maintaining productivity.^5–8^

Biostimulants are compounds derived from organic matter and microorganisms that, when applied to crops, enhance nutrient uptake, stimulate plant growth, and improve plant quality.^4,9^ Among the materials associated with biostimulant effects, humic substances (HS) stand out, with their fractions composed of humic and fulvic acids, derived from the decomposition of plant and animal residues.^10–12^

The effects of HS include the activation of enzymes involved in carbon and nitrogen metabolism,^13^ as well as photosynthesis and protein synthesis, leading to increased growth, higher chlorophyll content, root development, and improved nutrient uptake.^4,13^ These benefits are attributed to the presence of phytohormones such as indole-3-acetic acid, abscisic acid, and cytokinins, which, in addition to promoting growth, also play a role in plant adaptation to biotic and abiotic stresses.^14,15^

HS can be applied both to the soil and via foliar application, the latter being a complementary method of soil fertilization.^16^ Studies also reveal that HS exhibit high affinity for metal ions, mainly due to their strong complexation capacity.^17,18^ These properties can positively influence plant growth by protecting roots from leaching and increasing the availability of poorly soluble micronutrients for plant uptake.^19,20^

Foliar fertilization using HS has demonstrated significant agronomic benefits in various crops, leading to increased yield and grain size in maize (*Zea mays*), enhanced leaf area, protein concentration, and growth in lettuce (*Lactuca sativa*), higher productivity of garlic (*Allium sativum* L.) and tomato (*Solanum lycopersicum* L.).^13,21–23^ Building upon these promising outcomes, recent research has increasingly focused on alternative humified organic materials derived from agro-industrial residues, particularly those exhibiting biostimulant properties. Among these, humic-like substances (HLS) have gained prominence due to their potential to enhance crop performance while contributing to biomass valorization.^24,25^ These materials can be produced through processes such as composting,^26^ pyrolysis,^27^ hydrothermal humification,^15,25^ and hydrothermal carbonization (HTC).^28–30^

The presence of HLS in carbonaceous materials derived from lignocellulosic biomass has shown similarities to HS, especially in studies of complexation and interactions with metals,^29,30^ promotion of photosynthesis,^24^ and effects on corn germination.^25^ Other studies have observed that HLS also contributed to the increase in carbon content, improvement in biological activity, availability of nutrients, acceleration of seed germination, and resistance to abiotic stress.^15,31,32^

HTC's process of residual biomass, such as sugar cane bagasse and vinasse, using acid and basic additives, has been studied for the production of hydrochar (HC) and nutrient immobilization.^33–35^ Some studies have investigated the behavior of HLS extracted from HC in metal complexation and germination tests.^30,36^ The bioactive effect of HLS extracts from hydrochars promoted the elongation of coleoptiles and lateral roots in the growth of *Zea mays*, highlighting the phytohormonal stimulation potential of these humic-like materials.^36^

Despite the increasing interest in HLS, most studies to date have been restricted to soil applications. The use of HLS via foliar fertilization, especially those obtained from hydrochars produced with sugar cane bagasse and vinasse, remains underexplored.^16,37,38^ Foliar fertilization may offer faster nutrient uptake and reduce losses by leaching or volatilization,^39,40^ contributing to improved nutrient assimilation efficiency.^40^

In this context, the present study investigated the biostimulant potential of HLS extracted from hydrochars derived from sugar-energy industry residues and applied via foliar fertilization in tomato (*Solanum lycopersicum* L.) cultivation. The HLS were obtained from hydrochars produced in acidic and alkaline reaction media, chemically characterized, and tested both individually and in combination with a commercial fertilizer. Plant growth, dry biomass, chlorophyll content, and leaf micromorphology were evaluated to assess the treatment efficacy. This approach aimed to reduce reliance on finite fertilizer resources while enhancing the valorization of agro-industrial residues within a circular economy framework.

## 2 Materials and Methods

### 2.1 Reagents and Biomass Sources

The sugar cane bagasse and vinasse used in the HTC process were provided by a sugar-energy industry located in the São Paulo state, Brazil, and the project is registered in the National System for the Management of Genetic Heritage and Associated Traditional Knowledge (SISGEN) under the number A0018C2. The sugar cane bagasse was air-dried, ground, homogenized, and sieved (<0.5 mm), while the vinasse was collected and stored under refrigeration at −18 °C. Prior to use, it was thawed and homogenized by agitation.

### 2.2 Hydrochar Production and Humic-like Substance Extraction

Two HCs were produced through the HTC process of sugar cane bagasse and vinasse, resulting in HC_A (with H_3_PO_4_ as an additive) and HC_B (with KOH as an additive), according to the method proposed by Melo et al.^33^ In this method, sugar cane bagasse and vinasse were added to a stainless-steel reactor in a 1:20 (w/v) ratio, with the addition of 4.0% phosphoric acid for HC_A and 10 mL of a 4.0 mol L^–1^ potassium hydroxide solution, leading to a final concentration of 0.1 mol L^–1^ in the reaction for HC_B. The reagents used for the preparation of the solutions were potassium hydroxide (Sigma-Aldrich, 85%) and phosphoric acid (Sigma-Aldrich, 85%), employed as additives in the hydrothermal carbonization reactions.

For each condition, the reactor was heated to a temperature of 230 ± 10 °C in a muffle furnace for 13 h. The solid (HC) was separated from the liquid phase (process water) by filtration and washed with distilled water until a constant pH. The HC was dried at 65 °C for 24 h, homogenized, and stored.

HLSs were extracted from the HCs following the procedure described in the literature.^30,36^ HLSs were extracted from HC_A and HC_B, and were designated HLS_A and HLS_B, respectively. For this, 1.0 g of HC was used for every 10.0 mL of KOH (0.1 mol L^–1^). This solution was stirred for 4 h under a nitrogen flow (N_2_). The samples were centrifuged at 3,300 rpm for 15 min. Four successive extractions were performed until the final extract showed a lighter color compared to the previous extracts.^41^ Subsequently, the extracts were lyophilized using a MODULYO D-23 lyophilizer (Thermo Electron Corporation, United Kingdom).

### 2.3 Characterization of Hydrochars and Humic-like Substances

The C, H, and N contents in the HC and HLS were determined using an elemental analyzer, Fisons, model EA 1108 (United States of America). The ash percentage was obtained from thermogravimetric analysis curves using a PerkinElmer thermogravimetric analyzer, model TGA-4000 (United States of America) under a synthetic air atmosphere, with stabilization for 1 min at 30 °C and a heating ramp up to 900 °C at 10 °C min^–1^.

The FTIR spectra were obtained in a PerkinElmer model SpectrumTwo (Australia) in the range of 4000–400 cm^–1^ with a resolution of 4 cm^–1^. Molecular fluorescence spectra were obtained from HLS solutions at 5.0 mg TOC L^–1^ (Figure S1) in NaHCO_3_ (0.05 mol L^–1^, pH 8.0) using a spectrofluorimeter Varian, model Cary Eclipse, (Australia) in the excitation–emission matrix (EEM) mode, varying the excitation and emission ranges from 200 to 600 nm, with a slit width of 5 nm, scan speed of 1200 nm min^–1^, and detector voltage of 700 V.

The solid-state ^13^C NMR analysis was performed using the Cross-Polarization Magic Angle Spinning (CPMAS) technique on a Bruker spectrometer, model Avance III HD 400WB (United States of America) equipped with a 4.0 mm probe, a spinning frequency of 10 kHz, a relaxation time of 1 s, a contact time of 1 ms, an acquisition time of 20 ms, and 4000 scans. The ^13^C NMR spectra were processed using TopSpin 3.7 software (Academic License, Bruker), and the following chemical shift regions were defined: C-alkyl (0–45 ppm), C-methoxy or C-alkyl-N (46–60 ppm), O-alkyl-C (60–110 ppm), alkyl/aromatic or unsubstituted C (110–145 ppm), O-substituted aromatic C (145–160 ppm), and carboxyl C (160–190 ppm). The area of each spectral region was normalized by the sum of all areas to obtain the relative area. To assess structural differences, dimensionless indexes were calculated based on the relative areas: aromaticity index (AI) and hydrophobicity index (HI). The HI was calculated by the ratio of the integrated relative areas of hydrophobic carbons (0–45 + 110–160 ppm) to hydrophilic carbons (45–60 + 60–110 + 160–190 ppm) and AI was determined by the ratio of the integrated areas of aryl groups (110–160 ppm) to alkyl groups (0–45 + 60–110 ppm).^36,42^

### 2.4 Tomato Growth Assays: Foliar Application of Humic-like Substances in the Presence and Absence of Nutrients

Foliar fertilizer application experiments were conducted with eight treatments, with *n* = 5 and *n* = 4 for the assays with HLS_A and HLS_B, respectively (Table S1). Tomato plants (*Solanum lycopersicum* L.) were grown in a climate chamber (TE-4002, Tecnal, Brazil) under the conditions of 26 ± 2 °C, 55% relative humidity, and a 16/8 h (day/night) photoperiod for 35 days.

The assays included two control groups: one, T-WC, consisted of spraying only distilled water, and the other, T-FC, consisted of a commercial foliar fertilizer diluted 500:0.75 (v:v) (Titanium Completo - Solo Rico; composition: 5% N, 8% P, 5% K, 1% Ca, 0.6% Mg, 0.04% B, 0.02% Cu, 0.5% Mn, 1% Zn + 28% amino acids). Six additional application treatments were tested: three with HLS and three with a mixture of HLS and commercial fertilizer (CF). The HLS were applied at the following concentrations: 50 mg of TOC L^–1^ (T-50 and T-50F), 100 mg of TOC L^–1^ (T-100 and T-100F), and 300 mg of TOC L^–1^ (T-300 and T-300F) (Table S1). The pH of the HLS_A and HLS_B extracts was 12.69 and 13.50, respectively. Following the preparation of the final dilutions (50, 100, and 300 mg TOC L^–1^), the pH of the resulting solutions was adjusted, when required, using HCl (0.1 mol L^–1^) to bring the pH closer to neutral conditions.

Tomato seeds were commercially obtained (Sakata, 95% germination rate), and five seeds were sown per pot (13 cm height, 7 cm width) with 700 g of Ultisol soil at a depth of 2 cm. The Ultisol was collected from the rural area of Quatá, São Paulo, Brazil (20° 48′19.79”S, 49° 19′43.51”W), and its physicochemical properties are presented in Table S2.

After 12 days of sowing, seedlings were thinned, leaving only the best-developed one per pot, and NPK 20:20:20 fertilizer was applied to all treatments, including the control groups. Pots were irrigated with distilled water in a 50–100 mL volume at the beginning of the light period. The foliar application of the previously described treatments was carried out from a 30 cm distance using manual spraying, with three doses (∼700 μL each per pot) applied at 7-day intervals during the first three h of the light period.

Plant growth was monitored for 35 days, and the development was evaluated by measuring root length (cm) and shoot length (cm) using ImageJ software. The dry weight of both parts was determined after oven-drying at 105 °C until reaching a constant weight, and the results were expressed as dry weight in grams.

The chlorophyll index (Falker chlorophyll index–ICF) was measured using a chlorophyll meter (clorofiLOG, Falker, Brazil). The ICF was obtained as the average of three measurements from different points on the middle third of the same nonsenescent leaf, taken in the morning to minimize light-induced variations.^21^

For the morphological analysis of tomato leaves after exposure to HLS for 35 days, leaf fragments were subjected to a scanning electron microscopy (SEM) analysis. The leaves were fixed in an aqueous solution containing 2.5% glutaraldehyde, 4.0% paraformaldehyde, and 0.05 mol L^–1^ sodium cacodylate buffer (pH 7.2) at room temperature. Thereafter, the samples were three-fold washed for 45 min in 0.05 mol L^–1^ sodium cacodylate buffer. Subsequently, the samples were postfixed in 1% osmium tetroxide in 0.05 mol L^–1^ sodium cacodylate buffer and dehydrated in a graded acetone series. The dehydrated samples were critical point dried, mounted on sample holders with double-sided adhesive tape, and coated with a 20 nm gold layer by sputtering. Observations were performed using a scanning electron microscope (Quanta 450 FEG/ThermoFisher) at 20 kV, employing secondary electron detection.

### 2.5 Data Analysis

The effects of HLS as a foliar fertilization additive on plant growth parameters and chlorophyll content were analyzed using one-way ANOVA, with *F*-tests for significance (*p* < 0.05) and Tukey’s *post hoc* test for mean comparisons (α = 0.05). When normality and homoscedasticity assumptions were not met, the nonparametric Kruskal–Wallis test was applied (root length, shoot length), followed by Dunn’s test for pairwise comparisons (α = 0.05). To fit the root dry weight data to the statistical analysis, the data were transformed into BoxCox. In addition, an exploratory analysis of the obtained growth data set was conducted using principal component analysis (PCA) based on the correlation matrix. All analyses and graphics were produced using OriginPro 2024 (OriginLab, Northampton, MA, United States of America).

## 3 Results and Discussion

### 3.1 Compositional and Structural Investigation of HCs and HLSs

The characteristics of the materials produced during the HTC process and extraction are already described in the literature.^30,34,36^ However, due to the heterogeneity of the byproducts used and the complexity of the lignocellulosic raw materials, new characterizations were performed to ensure the reproducibility of the obtained data.^33,43^ Table 1 shows the elemental composition of C, H, N, ash, and atomic ratios of the HCs and HLS.

**Table 1:** CHN Elemental Composition, Ash Content, and H/C, C/N and Atomic Ratios of HC_A and HC_B and Their Respective and HLS

	elemental composition			ash	atomic ratios	
samples	C (%)	H (%)	N (%)	(%)	H/C	C/N
HC_A	59.73	6.22	2.38	23.18	1.25	29.28
HC_B	57.26	5.97	2.90	21.24	1.25	23.03
HLS_A	56.87	6.19	3.57	21.60	1.30	18.84
HLS_B	57.16	5.83	3.88	6.20	1.22	17.17

Two HCs and HLS_A and HLS_B (Table 1) presented results that agree with the literature.^30,34^ C and H contents showed similar values, which are characteristic of hydrochars.^34^ The ash content of HC_A and HLS_A was higher than that produced with KOH additive HC_B and HLS_B (Table 1 and Figure S2).

The N amount was 3.57% for HLS_A and 3.88% for HLS_B, which are similar values compared to those reported for HS (N: 3.3%),^29^ and compared com values found for the same HS as well as for humic materials extracted from anthropogenic soil (Amazonian dark earth), which showed N content of 3.63% ± 0.04.^36^ H/C atomic ratio did not differ between HC_A and HC_B obtained in acidic and alkaline media. However, an unsaturation increase was observed for HLS_B (1.30) compared to that for HLS_A (1.22). The decrease in the H/C atomic ratio is associated with higher unsaturation content, indicating a higher degree of material aromatization.^44^ C/N ratio indicates the loss or gain of nitrogen groups. Accordingly, higher incorporation of N (atomic ratio C/N of 17.17) was observed in the HLS_B, which corroborates the results reported by Moura et al.,^30^ who used the same HTC reaction parameters with the addition of KOH, indicating atomic ratio values (C/N: 15.8).

The FTIR spectra (Figure 1) exhibited similar profiles with regard to the bands assigned to HCs and HLSs. The broad band in the 3500–3000 cm^–1^ region (Figure 1) was associated with O–H stretching of alcohols, phenols, and carboxylic acids, being more prominent in two HLSs. The bands between 2900 and 2850 cm^–1^ were attributed to C–H stretching of the aliphatic groups. Additionally, the band in the region of 1700 cm^–1^ was assigned to C=O stretching of carboxylic acids, aldehydes, and ketones, while the bands in the 1680–1600 cm^–1^ region were attributed to C=C stretching of aromatic rings.^45–47^

**Figure 1:**
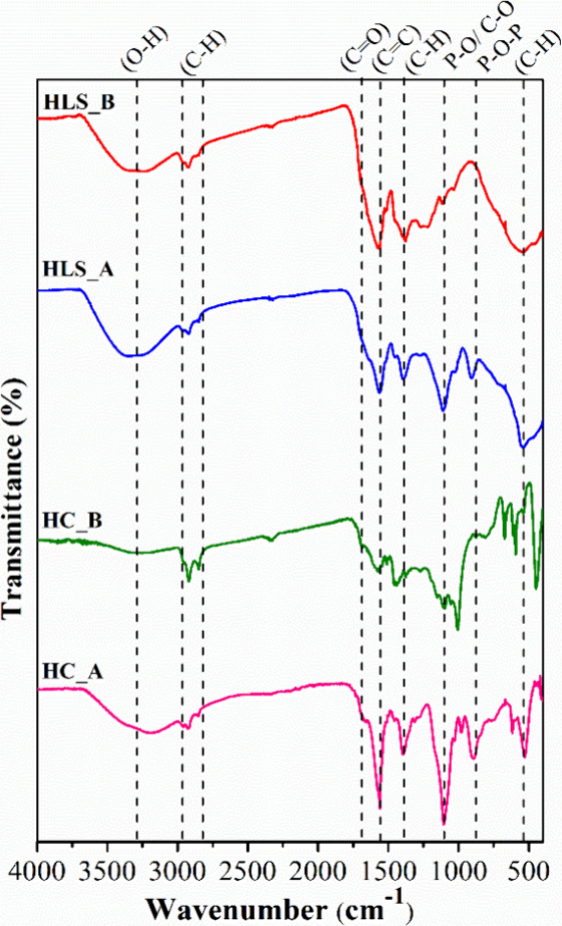
FTIR spectra of HC_A, HC_B, HLS_A, and HLS_B.

The band in the region of 1400 cm^–1^ was associated with C–H deformation, while the 1140–770 cm^–1^ range corresponded to overlapping bands related to C–O–C deformations. Specifically for HLS_A and HC_A, the vibrations at 1110–1050 and 900 cm^–1^ were associated with P–O and P–O–P stretching, respectively. The presence of these bands can be related to the addition of phosphoric acid during HTC.^48,49^ Notably, the bands mentioned above were absent in HC_B and HLS_B, as the additive used in the HTC process was KOH.

The HCs also differed considerably, as observed in the HC_B spectrum, by the absence of characteristic bands and less defined in the ranges of 3500–3000 and 1710–1700 cm^–1^, respectively, as well as a more pronounced band at 1028 cm^–1^. These results suggest that the addition of KOH during the HTC process promoted a significant degradation of phenolic groups, carboxylic acids, and their derivatives, consistent with similar studies.^50,51^

Furthermore, HLS exhibited functional groups similar to the ones present in HS from Amazonian Dark Earth, although the HS displayed more pronounced aromatic domains.^29^ In comparison, HLS in this study showed major functionalization than the artificial humic acids from bovine manure studied by Sarlaki et al.^47^

Fluorescence spectra in the EEM mode of the HLS are presented in Figure 2. The fluorescence emission regions can be attributed to different types of fluorophores, with emissions in the blue region (300–400 nm) being characteristic of fluorophores with major aliphatic character and less π-π conjugation. In comparison, fluorophores that emit in the region (500–600 nm) are associated with the presence of compounds with major π–π conjugation, such as aromatic compounds.^52^

**Figure 2:**
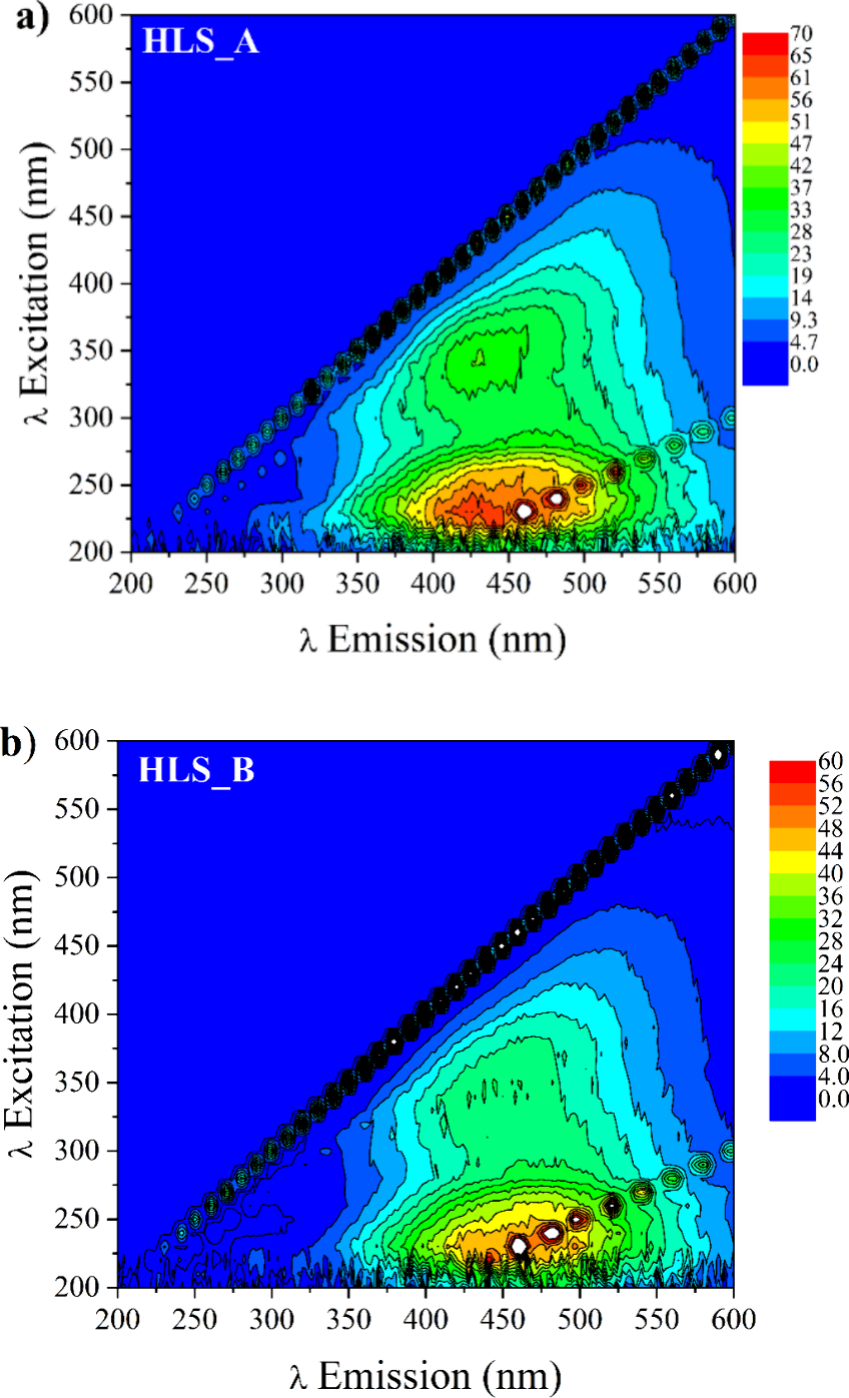
Fluorescence spectra in the EEM mode of the HLS solutions at 5.0 mg TOC L^–1^: (a) HLS_A and (b) HLS_B.

In this context, HLS_A (Figure 2) exhibited two maximum emission peaks: a more intense one at λEx/Em 230/430 nm and another at λEx/Em 340/430 nm, both characteristic of fluorophores classified as humic-like.^53–55^ For HLS_B, the most intense peak was observed at λEx/Em 230/450 nm, with a slight redshift in the emission, characteristic of humic materials. This behavior suggests the presence of fluorophores with greater conjugation in their composition, corroborating the FTIR analysis, which showed greater evidence in the C=C band for this material, although the spectral differences are subtle.^29,56^

The ^13^C NMR spectra obtained for the HC and HLS, as well as the relative percentages of the C distribution, are presented in Figure 3 and Table 2, respectively. Both for HC and HLS, the NMR spectra revealed a complex molecular composition. This observation was also made in other studies with humic substances.^56,57^

**Figure 3:**
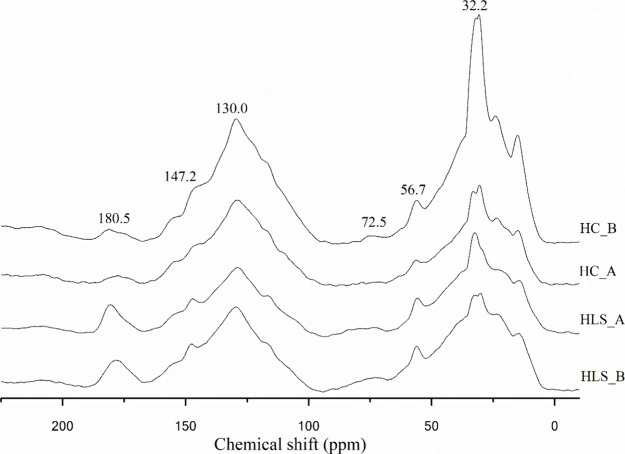
^13^C NMR spectra of HC_A, HC_B, HLS_A and HLS_B.

**Table 2:** Integrated Relative Areas (%) of the Chemical Shift Regions (ppm) in the ^13^C NMR Spectra and Structural Indices of HC and HLS

	chemical shift (ppm)						structural index	
	carboxylic acid (δ190–160)	O-aryl (δ 160–140)	C-Aryl (δ 140–110)	O-Alkyl (δ 110–60)	O–CH_3_ (δ 60–45)	alkyl (δ 45–0)	HI^a^	AI^b^
HC_A	0.07	9.96	35.11	5.83	7.18	41.86	3.77	0.95
HC_B	–^c^	6.87	31.52	3.53	6.98	53.10	5.26	0.68
HLS_A	2.80	8.73	28.16	0.10	9.63	50.58	4.11	0.73
HLS_B	5.76	10.31	27.33	8.28	8.98	39.35	2.31	0.79

a Hydrophobicity index (HI) = Σ (0–45) + (110–160)/Σ (45–60) + (60–110) + (160–190)].

b Aromaticity index (AI) = [(110–160)/Σ (0–45) + (60–110)].

c (−) Not detected.

The chemical shifts between 0 and 45 ppm were attributed to methylene and methyl groups in the alkyl chains of hydrocarbons formed during biomass carbonization. The peak at 72 ppm corresponds to O-alkyl-C (60–110 ppm region) and is attributed to the pyranoside structure.^36^

The chemical shifts between 110 and 140 ppm (C-Aryl) were assigned to unsubstituted and alkyl-substituted carbons in condensed aromatic fractions, suggesting the occurrence of aromatization reactions.^30^ In both the HC and HLS cases, this indicates the formation/extraction of similar molecular structures. The region between 140–160 ppm (O-Aryl) refers to the presence of aromatic carbons linked to oxygen, such as phenolic and methoxyl groups, as well as those identified in other humic-like materials extracted from HC with biomass from the sugar-alcohol industry.^29,30^

In the 160–190 ppm region, the HLS spectra presented peaks at 180.5 ppm, slightly more intense than those in the HC spectra. This signal is associated with carbons from carboxylic acids or esters.^58^

Table 2 presents the relative integrated areas (%) along the chemical shifts (ppm) in the ^13^C NMR spectra, as well as the hydrophobicity (HI) and aromaticity (AI) indexes of the HC and their respective HLS. The HI index is commonly related to the degree of stabilization of organic matter. High values indicate an increase in the stabilization process of organic matter, associated with an increase in hydrophobic compounds. On the other hand, the AI ratio is used to assess the proportion of aromatic structures in the structural properties of humified materials.^29,36^

In this regard, there is a predominance of the relative percentages of C-alkyl, C-Aryl, and O-Aryl, also observed for other humic-like substances derived from HC.^29^ The HLS_B sample is slightly more aromatic (AI = 0.79) and less hydrophobic (HI = 2.31) than the HLS_A sample, corroborating the lower atomic ratios and the fluorescence profile (Table 1 and Figure 2). The extraction of HLS from the HC resulted in an increase in carboxylic acid groups (190–160 ppm) and a reduction in C-aryl (140–110 ppm).

The aromaticity and hydrophobicity indexes (Table 2) highlight the structural differences between the HLS. Although the AI values were similar, HLS_A showed a higher HI compared to HLS_B, due to the different additives used in the HTC. The lower HI value indicates an increase in hydrophilic groups, as well as higher values in the chemical shift of carboxylic acids (190–160 ppm) and O-aryl (160–140 ppm).^30^

### 3.2 Effects of HLS in Foliar Fertilization on Plant Growth

The stimulating effects resulting from the foliar applications of HLS and their effects in combination with the commercial fertilizer (CF) on the development of tomato plants (*Solanum lycopersicum* L.*)* were observed (Figures 4 and 5 and Tables S3–S5).

**Figure 4:**
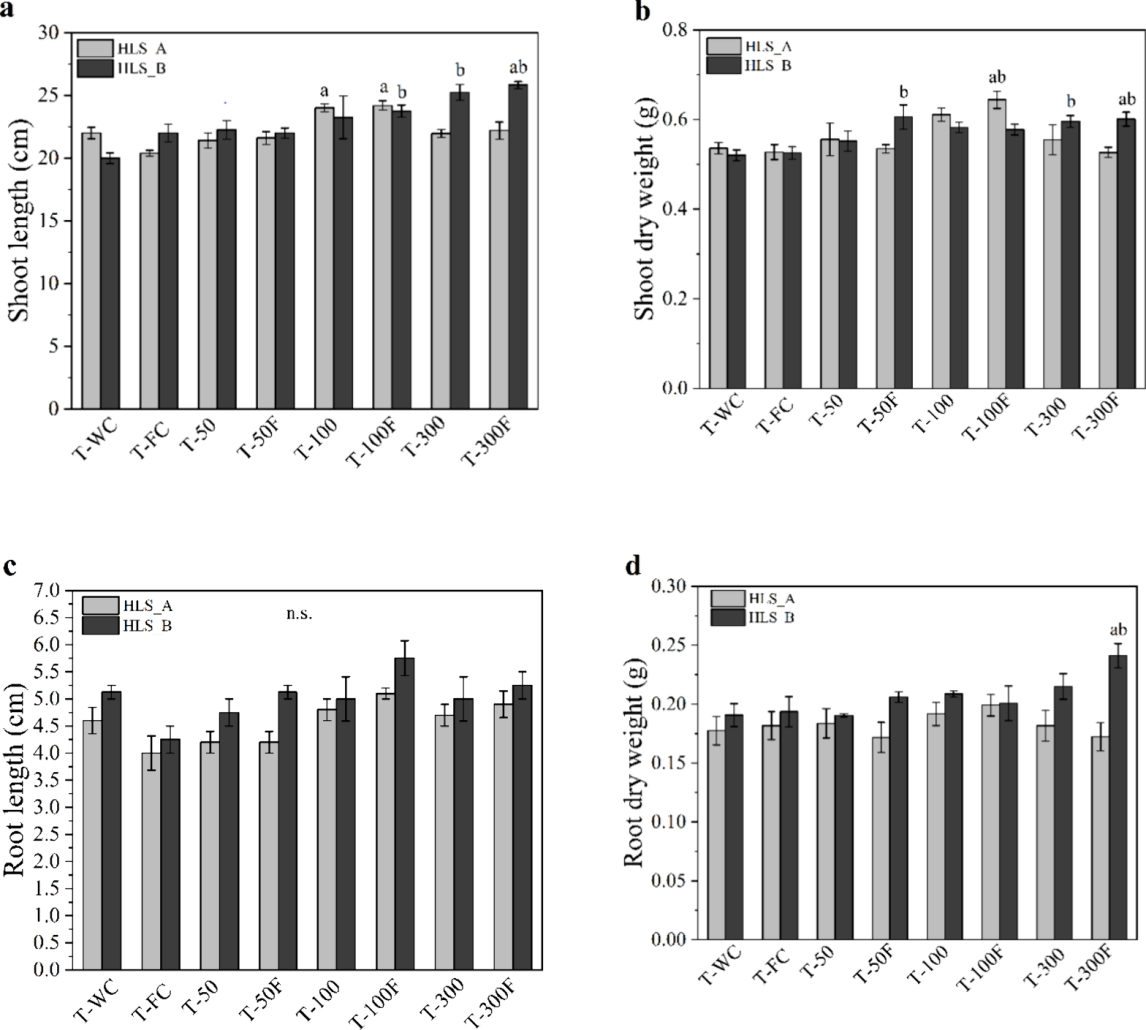
Plant growth response for root and shoot to foliar application of different HLS rates after 35 days: (a) shoot length; (b) shoot dry weight; (c) root length, and (d) root dry weight. Columns represent mean values ± standard deviation (SD). Letters above the bars denote significant differences between treatments and controls, based on mean comparison tests (α = 0.05), where ‘a’ indicates significant differences from the commercial fertilizer control (T-FC), and ‘b’ indicates differences from the distilled water control (T-WC) for *p* ≤ 0.05, and “n.s.” denotes no statistical differences between treatments.

**Figure 5:**
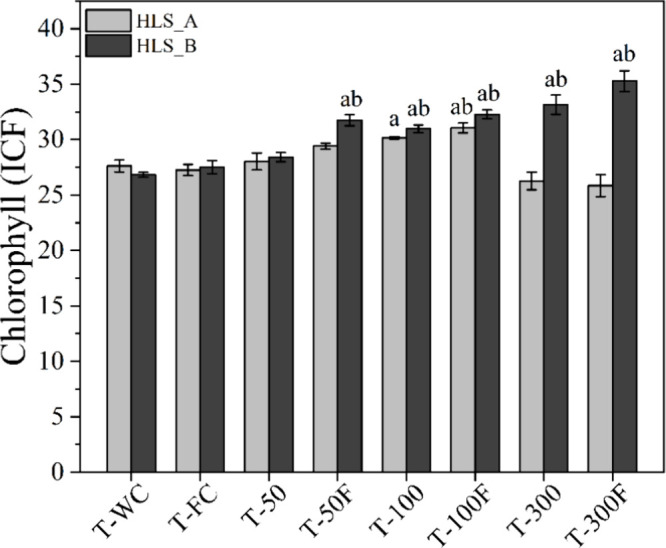
Effect of the foliar application of HLS on the total chlorophyll index (ICF) of tomato leaves after 35 days: a) HLS_A (*n* = 5) and b) HLS_B (*n* = 4). Columns represent mean values ± SD. Letters above the bars denote significant differences between treatments and controls, based on mean comparison tests (α = 0.05), where ‘*a*’ indicates significant differences from the commercial fertilizer control (T-FC), and ‘*b*’ indicates differences from the distilled water control (T-WC) for *p* ≤ 0.05.

In the shoot (leaves + stem) of the plants (Figure 4a,b), the length was significantly influenced by the application of both HLS (HLS_A: χ^2^=24.183; df = 7, *p* = 0.00106; and HLS_B: *F*_7;24_ = 5.767; *p* = 0.00053), but at different application rates (Figure 4a and Table S3). In the experiment with HLS_A, the T-100F treatment resulted in a significant increase of approximately 18% in growth compared with the control with CF, highlighting the beneficial effect of the association with the commercial foliar fertilizer. The stimulating effect of HLS_A alone was also observed in T-100 treatment. For HLS_B, the T-300F treatment resulted in an average shoot length of 25.8 ± 0.57 cm, significantly greater than the controls, with increases of 29.1% compared to T-WC and 17.4% compared to T-FC (Figure 4a and Table S4).

Comparable improvements in shoot length (approximately 28.3%) have been reported in tomato plants following foliar application of Ecohume, a commercial humic biostimulant.^23^ Moreover, under abiotic stress conditions, modest increases in shoot length (5.2%) were observed after foliar application of fulvic acid in tomato plants,^59^ indicating the general responsiveness of this crop to humified substances.

For the dry weight of the shoot (Figure 4b), there were also significant positive effects for the same treatment rates that influenced the length of the shoot (HLS_A: *F*_7;32_= 4.007; *p* = 0.00298 and HLS_B: *F*_7;23_ = 4.320; p = 0.00349). In HLS_A, the T-100F treatment resulted in a dry biomass of 0.64 ± 0.04 g, representing an increase of approximately 20% compared to the T-WC (0.54 ± 0.03 g) and T-FC (0.53 ± 0.04 g) controls. On the other hand, the application of HLS_B in the T-300F treatment also promoted a significant increase, though smaller than the one observed with HLS_A, reaching 0.60 ± 0.03 g (∼15%). These results highlight the beneficial effect of HLS when added to CF in increasing the dry biomass. Additionally, it can be observed that for HLS_B, the T-300 (without CF) treatment also showed positive results compared to T-WC, emphasizing the stimulating potential of HLS_B even when applied alone (Figure 4b).

However, the foliar dry weight gain was lower compared to that observed in cucumber plants (*Cucumis sativus* L.) subjected to the foliar application of humic substances, in which the dry mass increased by 46%.^37^ These variations may be attributed to differences between plant species.^23,59^

In the root evaluation, although no significant difference was detected between the mean lengths (*p* > 0.05), there was a tendency for greater lengths in the treatments with T-100F (HLS_A 5.1 ± 0.2 cm and HLS_B 5.7 ± 0.6 cm) and T-300F (HLS_A 4.9 ± 0.5 cm and 5.25 ± 0.5 cm) for both HLS (Figure 4c). However, it is worth highlighting that the median concentrations tested (100 mg of TOC L^–1^) of HLS resulted in better stimuli in root length. For dry biomass, the 300 mg TOC L^–1^ rate of HLS_B again stood out as the only treatment that also promoted a significant increase in the biomass parameter (HLS_B: *F*_7;23_ = 3.267; *p* = 0.01469). The combination of HLS_B with CF resulted in an increase of 26.4 and 24.5% compared with the T-WC and T-FC controls, respectively (Figure 4d).

These results demonstrate that the foliar application of HLS derived from hydrochar is effective, capable of stimulating plant growth, both in the shoot and roots, but with different application rates depending on the reaction of hydrochar production. In this sense, the synergistic effect of HLS_B with CF stands out, which promoted similar results to HLS_A for the shoot but also promoted stimulation of the roots.

Many studies have explored the effect of HS application in soil and described possible mechanisms of its stimulating action on plants.^60–62^ The most accepted mechanisms for stimulating plants via roots are based on the increase in proton pump activity in the plasma membrane (PM) through the stimulation of genes encoding the PM H^+^-ATPase and other enzymes involved in nutrient absorption (i.e., nitrate reductase, glutamine synthetase); increased ionic transport; in addition to the possible stimulation through phytohormonal substances, with the presence of indoleacetic acid (IAA) and other compounds with cytokinin-like and gibberellin-like actions.^16^

Some differences in the effects of root and foliar applications were observed by Hita et al.,^37^ who evaluated both types of humic substances (HS) applications (40 mg TOC L^–1^) in cucumbers (*Cucumis sativus* L. var. *Ashley*) over a 24- to 72-h period. In that study, foliar HS application increased root and shoot growth, an effect also observed in the present work. It was also found that foliar application did not induce an increase in H^+^-ATPase activity or abscisic acid (ABA) levels. However, increases in indoleacetic acid (IAA), cytokinins, jasmonic acid (JA), and jasmonate of isoleucine were observed in roots and leaves, and salicylic acid (SA) was observed only in the leaves. It is well-known that JA and SA activate responses to stress conditions (such as pathogens and herbivores). Thus, the hormonal effect of HS (via foliar application) may be mediated by a transient stress trigger since its application does not simulate a natural environmental occurrence.

### 3.3 Response of Chlorophyll and Micromorphological Structures to HLS Foliar Application

The photosynthesis process is essential for plant development and crop yields, as the absorption of light energy and the conversion of CO_2_ are directly linked to plant growth.^63^ The present study evaluated chlorophyll through the ICF index. The application of HLS_A significantly influenced the chlorophyll index, ICF (*F*_7;32_ = 8.94; *p* < 0.0001) (Figure 5). The average ICF values in the control treatments of the experiment with HLS_A were 27.6 ± 1.20 and 27.2 ± 1.14 for T-WC and T-FC, respectively. For HLS_A applications, the T-100F treatment (31.0 ± 1.01) resulted in an increase in ICF by 12.4 and 13.9% compared to T-WC and T-FC, respectively, corroborating the results observed for growth parameters (Figure 4). As observed for other parameters, the application of HLS_A at the concentration of 300 mg TOC L^–1^ did not differ from the controls (*p* > 0.05) (Table S5).

Spraying HLS_B influenced the increase of ICF in the plants for most treatments, with increasing values observed from T-50F (*F*_7;24_ = 25.4; *p* < 0.0001) (Figure 5 and Table S5). In the applications with only HLS_B (treatments without CF), rates of 100 and 300 mg of TOC L^–1^ provided an increase in chlorophyll stimulation, with significant positive differences compared to both controls. Additionally, the application of the highest rate (T-300) promoted an increase of 31.5 and 28.2% compared to T-WC and T-FC, respectively. Similar increase rates, up to 37.9%, were observed in the chlorophyll content (SPAD index) in a hydroponic maize study with the application of artificial humic acid (AHA).^25^

The T-100F and T-300F treatments, in which HLS_B was combined with CF (Figure 5), also showed positive and significant results compared to the controls, with increases of 17.3 and 28.2% compared to T-FC, respectively. It is worth noting that these treatments did not differ from their respective comparatives, where only HLS_B was sprayed (*p* > 0.05). This demonstrates the isolated effect of HLS_B on chlorophyll stimulation. Additionally, the association effect of the HLS_B + CF combination can be observed in the T-50F treatment, which had an increase of 18.3% compared to T-WC and 15.4% compared to T-FC, while its comparative treatment with only HLS_B, T-50, showed no significant variation was observed (*p* > 0.05).

Chlorophyll production in plants can be stimulated by environmental factors such as water, light, nutrients, and hormones, as well as being influenced by the intrinsic characteristics of each species.^64^ Consistent with our results, other studies reveal that foliar application of humified compounds can increase chlorophyll levels, as they influence transpiration and induce carbon and nitrogen metabolism, although the mechanisms remain uncertain.^65–67^

The influence of HS on stomatal opening via auxin was demonstrated by Russell et al.,^68^ and biostimulatory effects on the production of chlorophyll a and b, as well as on the SPAD index, were also observed in tomato cultivation with the application of humic fractions (AH and AF derived from composting) inoculated with bacteria.^32^ Tejada et al.^69^ observed that the application of vermicompost liquid leachates in foliar sprays increased tomato yield in terms of fruit nutritional quality. These results were associated with leachates rich in HS, which promoted increased absorption of nitrogen, phosphorus, and potassium as well as higher chlorophyll concentration.

Zhi et al.^24^ highlighted that the results of photosynthetic parameters seem to be related to the type of CO_2_ fixation in C3 (*Lactuca sativa*) and C4 (*Zea mays* L.) plants evaluated in their study. To investigate this, synthesized humic acids (AHA) (cyanobacteria/KOH/200 °C/48 h) were applied as substrates in hydroponic cultivation. In C4 plants, AHA directly promoted photosynthesis, improving energy capture and conversion, but without impacting chlorophyll synthesis. On the other hand, in C3 plants (*lettuce*), there was an increase in photosynthesis parameters due to the increased chlorophyll synthesis, corroborating what was observed in our study with tomatoes C3 plant (Figure 5).

HLS_B also promoted greater root development, which may have contributed to better absorption of essential nutrients such as N, P, and Mg, involved in chlorophyll synthesis (Figure 5).^70^ Additionally, nitrogen assimilation is known to be stimulated by plant growth regulators, such as humic and humic-like substances, which exert auxin-like activity and promote plant development, chlorophyll content, and nutrient uptake.^65^ The chemical characterization of HLS_B revealed a content of nitrogen-containing compounds of 18.84%, slightly higher than that of HLS_A (17.17), which may partially account for the greater stimulation of the photosynthetic activity. Although the commercial fertilizer applied contained 5% nitrogen, chlorophyll levels did not differ between treatments T-300 and T-300F (*p* > 0.05), suggesting that the observed effect is primarily attributable to HLS_B (Figure 5).

The superior performance of HLS_B compared to HLS_A may also be associated with its molecular characteristics. HLS_B (Figure 2b) exhibited a more intense fluorescence peak at λEx/Em 230/450 nm with a slight redshift, typical of humic substances with higher aromatic and conjugated structures. These structural features have been linked to increased biological activity, particularly in improving nutrient uptake.^71^ Therefore, the combination of a higher nitrogen content and more conjugated molecular structures may have facilitated improved interactions with plant tissues, resulting in a greater stimulation of chlorophyll synthesis compared to HLS_A

Fragments of the leaves subjected to scanning electron microscopy (SEM) did not show significant changes in their micromorphology, on both the abaxial and adaxial surfaces. The samples exhibited closed stomata with no signs of anatomical ruptures (Figure S3). However, it is worth noting that closed stomata in plants often act as an adaptation mechanism, minimizing water loss and reducing the transpiration rate, which helps protect the leaves from dehydration.^72^

The main difference observed was the apparent decrease in the number of stomata and the presence of trichomes (glandular and nonglandular) on the leaves treated with HLS_B at 300 mg TOC L^–1^, both in the isolated application and in combination with CF. This suggests that a regulatory mechanism may have occurred to mitigate stress occurrence, as trichomes play a role in plant defense and adaptation, protecting them against various abiotic and biotic stresses.^73,74^

In the control groups, stomata were present on both surfaces (arrows), displaying smooth periclinal walls and sinuous-shaped cells on the abaxial and adaxial surfaces (Figure S3a–d), with no evidence of precipitates in the T-WC treatment. In the T-FC treatment, the observed morphology was similar (Figure S3e–h); however, small precipitates were noted on both leaf surfaces (ellipses), likely resulting from the concentration of certain metal ions present in the commercial foliar fertilizer (CF). The leaves treated with HLS_A also showed an anatomy similar to that of the positive control T-FC, with the presence of precipitates in HLS_A exposed at 100 mg TOC L^–1^ in combination with CF (Figure S3m–p), while for the application with HLS_B (Figure S3q–x), no precipitates were observed.

### 3.4 Principal Component Analysis of Growth and Chlorophyll Data

Principal Component Analysis (PCA) of the sample distribution along the first two principal components (PC1 and PC2), which together explain the highest variance in the data set (85.9%), is presented in Figure 6a. PC1 accounts for 74.9% of the standardized total variance, with positive scores associated with biomass traits (shoot and root dry weight as well as chlorophyll content and shoot length), while PC2 represents 11.0% of the total variance, being strongly influenced by variations in root length.

**Figure 6:**
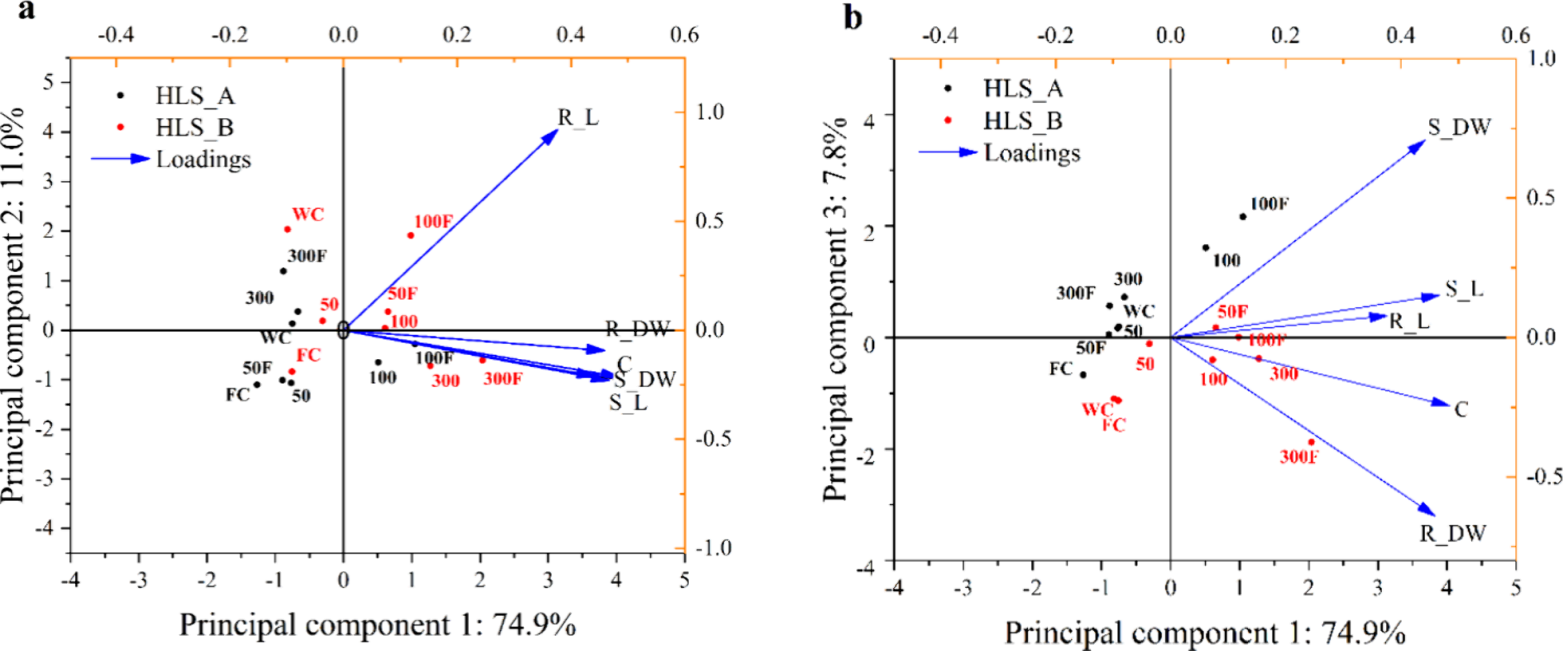
Principal component analysis: a) PC1 × PC2 and b) PC1 × PC3, based on tomato growth parameters (loadings: root length (R_L); root dry weight (R_DW); shoot length (S_L); shoot dry weight (S_DW) and Chlorophyll (C); points represent individual samples, colored by HLS type and treatment).

Thus, the main variations were reflected by the loadings in PC1, reinforcing that treatments T-100 and T-100F of HLS_A and T-300 and T-300F of HLS_B most strongly influenced the biomass, plant length, and chlorophyll content. Additionally, it was observed that as the concentration increased from 50 to 100F for HLS_B, they showed variable and positive scores, distanced from the controls, also demonstrating the stimulating effects of lower concentrations of HLS_B (Figure 6a and Tables S6 and S7). This behavior suggests that root length was affected by the increase in concentration, although it was not statistically significant, indicating that there may be an adaptive response in the roots at these concentrations, derived from the composition of HLS_B.

The standardized total variance explained by components 1 and 3 was 82.7% (Figure 6b). The clustering patterns indicate a separation between the application of HLS_A and HLS_B, as well as subtle differences between treatments with HLS alone and HLS + CF. For the concentrations that showed the greatest effects, HLS_A 100 mg of TOC L^–1^ and HLS_B 300 mg of TOC L^–1^, the scores were more positive for treatments with CF in both HLS (Table S7). For the best results in plant development, the application of HLS_A (T-100F) most influenced the biomass allocation to the shoot (leaves + stem), while HLS_B (T-300F) influenced root parameters. Furthermore, the influence of HLS_B + CF (50 and 100 mg of TOC L^–1^) on root length stimulation is evident.

Thus, the growth and development results of tomato plants observed in this study could be attributed to two main mechanisms: (i) the ability of humic-like substances (HLS) to interact with cations and (ii) the presence of phytohormone-like compounds. The complexing capacity of humic-like materials with metal ions has been demonstrated, primarily influenced by the presence of carboxylates.^30,45^ These properties can positively influence plant growth, mitigating the effects of leaching and increasing the availability of micronutrients that tend to form poorly soluble hydroxides, thereby facilitating their uptake by plants.^19,20,75,76^

The interaction of HLS_B with Cu (II) cations was described by Moura et al.,^30^ as well as the interaction of HLS extracted from hydrochar produced with acidic additive (H_2_SO_4_).^29^ These results support the potential of both HLS evaluated in this study, HLS_A and HLS_B, in binding to CF nutrients, making them more available. The high ash content in HLS_A, associated with the presence of inorganic compounds (Table 1), may have contributed to its performance, particularly in the T-100F treatment. On the other hand, HLS_B showed better results for T-300F and similar performance for T-300. These findings may be attributed to the presence of more oxygenated functional groups in this material (Table 2), which favor the complexation of nutrients, increasing their availability for plant absorption.

Studies indicate that HLS derived from lignocellulosic biomass can enhance seed germination and act as plant growth biostimulants.^25,77,78^ These positive effects are attributed to the presence of compounds with auxin- and gibberellin-like activity.^78^ HLS with a composition like that of this study stimulated the elongation of maize (*Z. mays*) seedlings.^36^ Therefore, the growth-promoting effect observed may also be attributed to the phytohormonal-like properties of phenolic compounds and lignin derivatives present in HLS (Table 2).

HLS, similar to natural HS, exhibits structural diversity due to its compositional characteristics. The solubility of their extracts may depend on their hydrophobic nature, which influences their bioactive properties. In this context, higher concentrations of more hydrophobic HLS did not stimulate the germination and growth of *Zea mays*, possibly due to the formation of aggregates in concentrated solutions.^36^

In our study, this trend was also observed, highlighting differences in the optimal concentrations for HLS_A and HLS_B. The more hydrophobic HLS_A (HI = 4.11) exhibited stimulatory effects only at intermediate concentrations (100 mg TOC L^–1^), while the less hydrophobic HLS_B (HI = 2.31) maintained its stimulatory effects even at higher concentrations (Table 2, Figures 5 and 6). The hydrophobicity of HLS varied according to the additive used in hydrochar production. The use of H_3_PO_4_ favored polysaccharide degradation in biomass, generating more polycondensed structures and more hydrophobic HLS. Moreover, HLS_B showed greater compositional diversity, with oxygenated structures and a higher N content than HLS_A, suggesting the presence of bioactive compounds.

In addition to these aspects, the difference in optimal doses between HLS_A (100 mg of TOC L^–1^) and HLS_B (300 mg of TOC L^–1^) can be attributed to their distinct molecular structures. HLS_B presented a higher degree of unsaturation (Table 1), indicative of greater aromatization and complexity.^44^ The bioactivity of humic substances is known to depend on molecular weight, hydrophobicity, and degree of aromatization, with more structurally complex materials often requiring higher concentration to induce physiological responses.^71,79,80^ In contrast, HLS_A, produced under acidic conditions, may contain more hydrophilic or smaller molecules that are active at lower concentrations. This aligns with the dose-dependent efficacy commonly observed for humic substances, where structural features influence the concentration required for optimal plant stimulation.^79^

## 4 Conclusions

The foliar application of humic-like substances extracted from hydrochars produced using sugar cane bagasse and vinasse demonstrated notable potential to enhance tomato growth and chlorophyll content. HLS_A promoted positive growth effects at the rate of 100 mg of TOC L^–1^, whereas HLS_B was most effective at 300 mg of TOC L^–1^, particularly in enhancing chlorophyll synthesis, a factor likely linked to its influence on additional growth parameters. Notably, the stimulatory effects of HLS_B were evident even in the absence of commercial fertilizer, reinforcing its intrinsic biostimulant activity. Additionally, the results obtained were comparable to those achieved with the commercial fertilizer (T-FC), highlighting the effectiveness of the HLS applications. These findings represent a promising advance in the circular economy by valorizing sugar-energy industry residues into high-value-added products. Future research should focus on field-scale evaluations over the full tomato cultivation cycle, assessing yield and productivity, as well as biochemical studies to elucidate the mechanisms involved in plant stimulation by HLS.

## Supplementary Material



## Data Availability

The data is available at the following address: https://hdl.handle.net/11449/296297

## Notes

Evaluation of the TOC concentration of HLS solutions for molecular fluorescence analysis; experimental design of foliar fertilization; physicochemical properties of the Ultisol; TG and DTG curves of all hydrochars and humic-like substances; tomato growth and chlorophyll data (mean ± SD), PCA loadings and scores, and SEM images of tomato leaves treated with HLS (PDF)

## Abbreviations

HLS: humic-like substances
HS: humic substances
TOC: total organic carbon
HTC: hydrothermal carbonization
HC: hydrochar
ICF: chlorophyll index
SEM: scanning electron microscopy
TGA: thermogravimetric analysis
FTIR: Fourier-transform infrared spectroscopy
EEM: excitation–emission matrix
CPMAS: cross-polarization magic angle spinning
NMR: nuclear magnetic resonance
AI: aromaticity index
HI: hydrophobicity index
PCA: principal component analysis
NPK: fertilizer containing nitrogen, phosphorus and potassium
IAA: indole-3-acetic acid
ABA: abscisic acid
JA: jasmonic acid
SA: salicylic acid
PM: plasma membrane.
