# Supplementary material for: Humic-like Substances Extracted from Hydrochar as an Additive for Foliar Fertilization in Tomato Plants
Source: ACS Omega. 2025 Aug 26;10(35):40133–45. doi: 10.1021/acsomega.5c05053 (PMC12423793; doi:10.1021/acsomega.5c05053)

Humic-like Substances Extracted from Hydrochar as an Additive for Foliar Fertilization in Tomato Plants

Suelen A. Mondek[a], Isabela C. Constantino[a], Odair P. Ferreira[b], Márcia C. Bisinoti[a], Emilio C. Miguel[c], Thaiz B. A. R. Miguel[d], Gustavo Metzker[a], Mauricio Boscolo[a], Altair B. Moreira[a*]

[a]Institute of Biosciences, Humanities and Exact Sciences, Department of Chemistry and Environmental Sciences, São Paulo State University (UNESP), Zip code 15054-000 São José do Rio Preto, São Paulo, Brazil.

[b]Department of Chemistry, Laboratory of Advanced Functional Materials (LaMFA), State University of Londrina (UEL), Zip code 86055-900, Londrina, Paraná, Brazil.

[c]Department of Metallurgical Engineering and Materials (DEMM) and Analytical Center, Biomaterials Laboratory (BIOMAT), Federal University of Ceará (UFC), Zip code 60440-900, Fortaleza, Ceará, Brazil.

[d]Food Engineering Department, Biotechnology Laboratory, Federal University of Ceará (UFC), Zip code 60440-900, Fortaleza, Ceará, Brazil

Corresponding author

[*]E-mail: altair.moreira@unesp.br

Tel: +55 17 3221-2509

Appendix A. Supplementary data

Figure S1. Evaluation of the TOC concentration of HLS solutions for molecular fluorescence analysis in the matrix emission-excitation (EEM) mode.

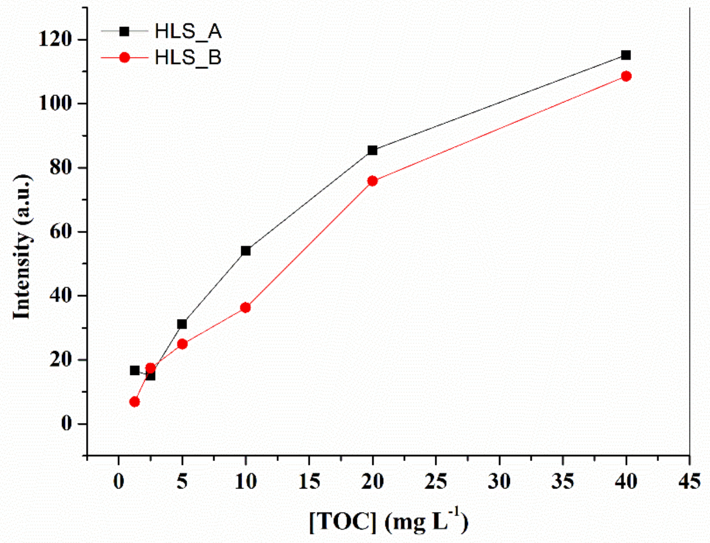

Table S1 - Experimental design of foliar fertilization treatments applied with HLS_A and HLS_B in the tomato cultivation.

ID	Treatment
T-WC	Control: distilled water with foliar application of 0 mg TOC L^{-1}
T-FC	Control: commercial fertilizer[1]
T-50	Only 50 mg TOC L^{-1} of HLS
T-50F	50 mg TOC L^{-1} of HLS + nutrients[1]
T-100	Only 100 mg TOC L^{-1} of HLS
T-100F	100 mg TOC L^{-1} of HLS + nutrients[1]
T-300	Only 300 mg TOC L^{-1} of HLS
T-300F	300 mg TOC L^{-1} of HLS + nutrients[1]

[1]Commercial fertilizer composition: 5% N; 8% P; 5% K; 1% Ca; 0.6% Mg; 0.04% B; 0.02% Cu; 0.5% Mn; 1% Zn + 28% amino acids.

Table S2. Physicochemical properties of the Ultisol used in the tomato plant growth experiment.

**Parameters**	**Content**
OM – organic matter (g dm^{-3})	18.7
pH ($CaCl_2$)*	4.8
OC – Organic Carbon (g dm^{-3})	10.9
B (mg dm^{-3})	0.2
Cu (mg dm^{-3})	1.7
Fe (mg dm^{-3})	60.0
Mn (mg dm^{-3})	19.5
Zn (mg dm^{-3})	6.4
CEC - Cation Exchange Capacity (mmol$_c$ dm^{-3})	47.7
Sum of Exchangeable Bases (mmol$_c$ dm^{-3})	21.7
Sand (total) (g kg^{-1})	880.0
Silt (g kg^{-1})	80.0
Clay (g kg^{-1})	40.0

* For growth experiments the pH was corrected with liming to pH 6.8.

Figure S2. Thermogravimetric Analysis a) HC_A, b) HC_B, c) HLS_A, and d) HLS_B.

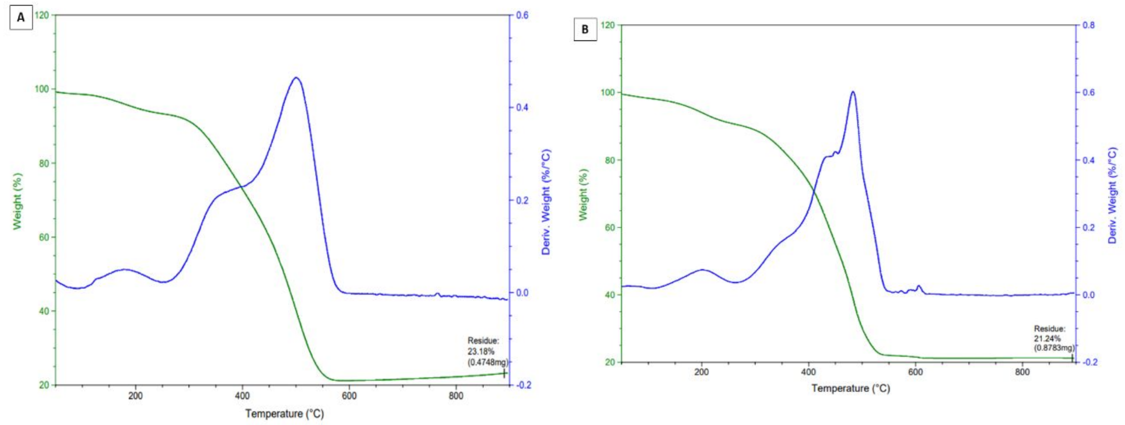

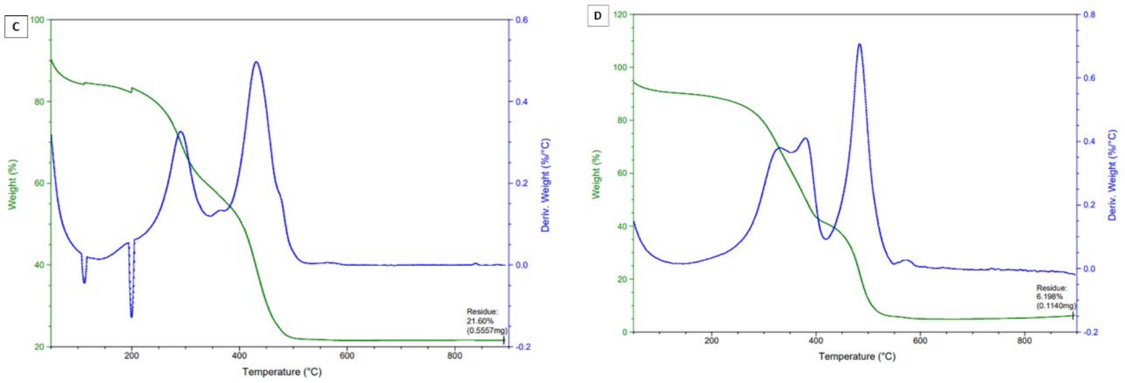

Table S3. Mean values ± standard error of mean (SD) obtained for tomato growth parameters in treatments applying HLS_A.

	Root Length(cm)	Shoot Length (cm)	Root Dry weight (g)	Shoot Dry weight (g)
	Mean±SD	Mean± SD	Mean± SD	Mean± SD
**T-WC**	4.60±0.55	22.00±1.00	0.177±0.027	0.536±0.029
**T-FC**	4.00±0.71	20.40±0.55	0.182±0.027	0.527±0.037
**T-50**	4.20±0.45	21.40±1.34	0.184±0.028	0.556±0.082
**T-50F**	4.20±0.45	21.60±1.14	0.172±0.028	0.535±0.021
**T-100**	4.80±0.45	24.00±0.71 a	0.192±0.022	0.611±0.033
**T-100F**	5.10±0.22	24.20±0.84 a	0.199±0.021	0.644±0.043 ab
**T-300**	4.70±0.45	21.96±0.71	0.182±0.030	0.555±0.075
**T-300F**	4.90±0.55	22.20±1.52	0.172±0.027	0.527±0.026
	(df=7; χ^2=15.786; p=0.02714)	(df=7; χ^2=24.183; p=0.00106)	($F_{7;32}$=0.966; p = 0.47189)	($F_{7;32}$=4.007; p = 0.00298)

Letters in each column denote significant differences between treatments and controls based on mean comparison tests (α = 0.05), where 'a' indicates a significant difference from the commercial fertilizer control (T-FC), and 'b' indicates a significant difference from the distilled water control (T-WC) for p ≤ 0.05.

Table S4. Mean values ± standard error of mean (SD) obtained for tomato growth parameters in treatments applying HLS_B.

	Root Length (cm)	Shoot Length (cm)	Root Dry weight (g)	Shoot Dry weight (g)

	Mean±SD	Mean± SD	Mean± SD	Mean± SD
**T-WC**	5.13±0.25	20.00±0.82	0.191±0.020	0.520±0.024
**T-FC**	4.25±0.50	22.00±1.41	0.194±0.022	0.526±0.028
**T-50**	4.75±0.50	22.25±1.50	0.190±0.003	0.552±0.045
**T-50F**	5.13±0.25	22.00±0.82	0.206±0.009	0.605±0.047 b
**T-100**	5.00±0.82	23.25±3.40	0.209±0.005	0.582±0.023
**T-100F**	5.75±0.65	23.75±0.96 b	0.201±0.030	0.578±0.024
**T-300**	5.00±0.82	25.25±1.26 b	0.215±0.022	0.596±0.026 b
**T-300F**	5.25±0.50	25.83±0.57ab	0.241±0.021 ab	0.601±0.031 ab
	(df=7; χ^2=11.936; *p*=0.10269)	($F_{7;24}$=5.767; *p* = 0.00053)	($F_{7;23}$=3.267; *p* = 0.01469)	($F_{7;23}$=4.320; *p* = 0.00349)

Letters in each column denote significant differences between treatments and controls based on mean comparison tests (α = 0.05), where ‘a’ indicates a significant difference from the commercial fertilizer control (T-FC), and ‘b’ indicates a significant difference from the distilled water control (T-WC) for p ≤ 0.05.

Table S5. Mean values ± standard error of mean (SD) obtained for Chlorophyll Index (ICF) in tomato leaves under treatments applying HLS_A and HLS_B.

**Treatments**	**Chlorophyll (ICF)**	
	**HLS_A**	**HLS_B**
	Mean±SD	Mean±SD
**T-WC**	27.62±1.20	26.83±0.46
**T-FC**	27.25±1.14	27.52±1.20
**T-50**	28.03±1.73	28.41±0.84
**T-50F**	29.41±0.62	31.75±0.99 ab
**T-100**	30.14±0.24 a	30.98±0.68 ab
**T-100F**	31.04±1.01 ab	32.27±0.81 ab
**T-300**	26.26±1.78	33.15±1.74 ab
**T-300F**	25.85±2.22	35.28±1.87 ab
	$F_{7;32}$=8.937; *p*<0.0001	$F_{7;24}$=25.412; *p*<0.0001

Letters in each column denote significant differences between treatments and controls based on mean comparison tests (α = 0.05), where ‘a’ indicates a significant difference from the commercial fertilizer control (T-FC), and ‘b’ indicates a significant difference from the distilled water control (T-WC) for p ≤ 0.05.

Table S6 - Loadings of Principal component analysis based on tomato growth parameters (R_DW: root dry weight; R_L: root length; S_DW: shoot dry weight; S_L: shoot length; C: chlorophyll index).

	**PC1**	**PC2**	**PC3**	**PC4**
**Percentage of variance**	**74.9%**	**11.0%**	**7.8%**	**4.2%**
	Loadings	Loadings	Loadings	Loadings
**R_DW**	0.45903	-0.0934	-0.64032	0.26153
**R_L**	0.37649	0.92104	0.07815	-0.03812
**S_DW**	0.44151	-0.21068	0.70766	0.47065
**S_L**	0.46671	-0.23042	0.15175	-0.8282
**C**	0.48455	-0.21326	-0.24508	0.15073

Table S7 - Scores of Principal component analysis based on tomato growth parameters (R_DM: root dry weight; R_L: root length; S_DW: shoot dry weight; S_H: shoot length; C: chlorophyll index).

		**PC1**	**PC2**	**PC3**	**PC4**
	**Treatments**	**Scores**	**Scores**	**Scores**	**Scores**
**HLS_A**	T-WC	-0.74989	0.13117	0.18868	-0.83244
	T-FC	-1.26838	-1.10017	-0.67192	0.91755
	T-50	-0.76591	-1.06522	0.16565	0.70062
	T-50F	-0.89418	-1.00781	0.05247	-0.30805
	T-100	0.50719	-0.6477	1.6131	-0.29024
	T-100F	1.04654	-0.27907	2.16502	0.68819
	T-300	-0.66771	0.37861	0.72666	-0.31877
	T-300F	-0.88175	1.19249	0.56976	-1.7426
**HLS_B**	T-WC	-0.81979	2.03479	-1.09525	1.19655
	T-FC	-0.75456	-0.83381	-1.13189	-0.54745
	T-50	-0.30628	0.18919	-0.11234	-0.18581
	T-50F	0.65368	0.37696	0.17795	2.39049
	T-100	0.60837	0.04095	-0.40174	0.37067
	T-100F	0.98419	1.91349	0.00494	-0.54186
	T-300	1.27174	-0.71822	-0.37702	-1.02371
	T-300F	2.03675	-0.60564	-1.87406	-0.47314

Figure S3. Scanning electron microscopy of tomato leaves exposed to different concentrations of HLS (mg TOC L^{-1}): **(a-d)**: T-WC; **(e-h)**: T-FC; **(i-l)**: HLS_A, T-100; **(m-p)**: HLS_A, T-100F, **(q-t)**: HLS_B, T-300 and , **(u-x)**: HLS_B, T-300F. Highlighted areas: stomata (arrow) and precipitates (ellipses). Scale Bars: a, c, e, g, i, k, m, o, q, s, u, w - 100 µm; and b, d, f, h, j, l, n, p, r, t, v, x - 20 µm.

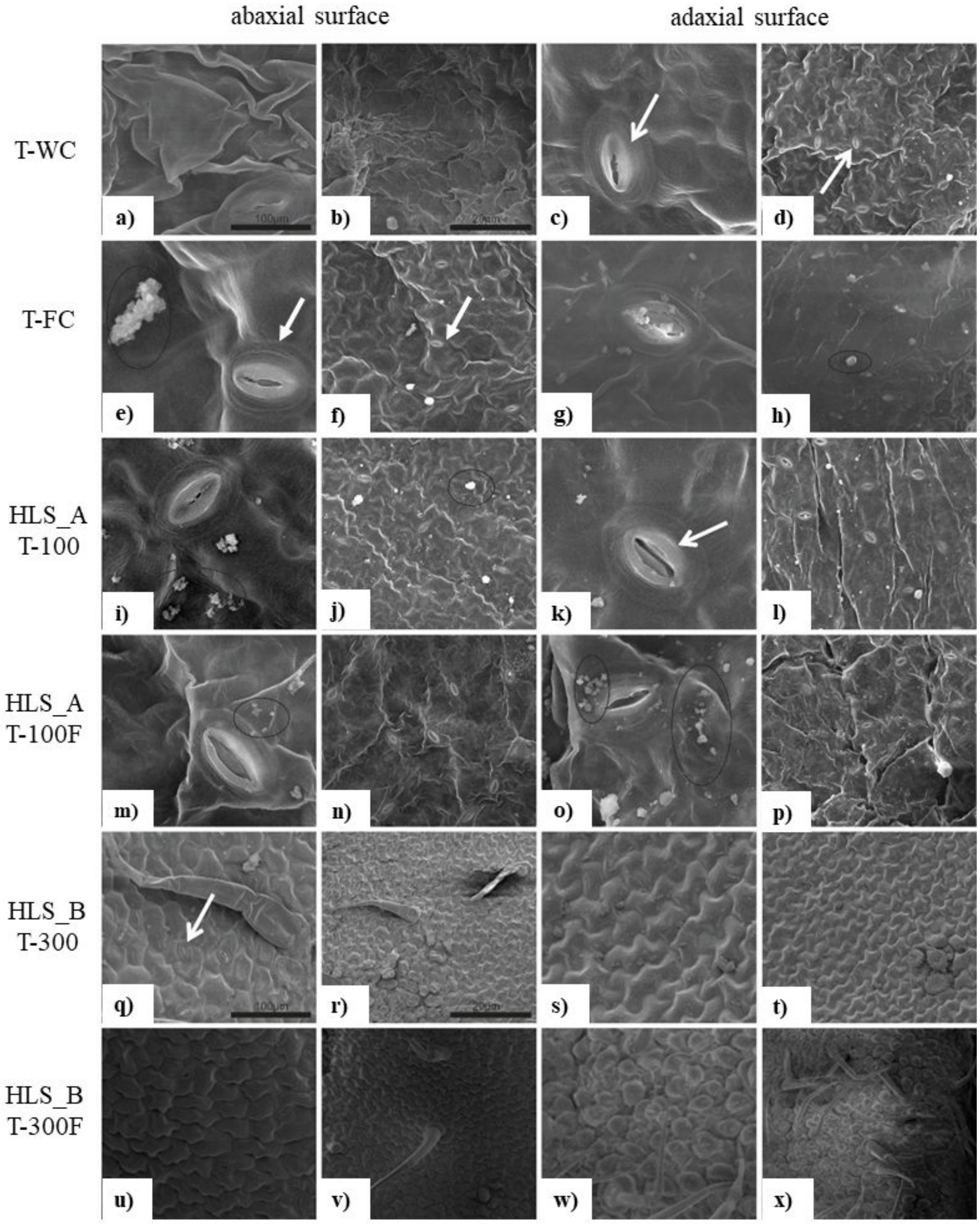

Supplement: Supplementary file 1 [file ao5c05053_si_001.pdf]
